# Collagen Turnover Is Associated with Disease Severity, Bone Marrow Fibrosis, and the *JAK2V617F* Variant Allele Frequency in Myeloproliferative Neoplasms

**DOI:** 10.3390/cancers18152529

**Published:** 2026-08-06

**Authors:** Caroline Norup Bistrup, Morten Kranker Larsen, Peter Junker, Vibe Skov, Lasse Kjær, Trine Alma Knudsen, Morten Karsdal, Nicholas Willumsen, Hans Carl Hasselbalch

**Affiliations:** 1 Nordic Bioscience A/S, 2730 Herlev, Denmark; mk@nordicbio.com (M.K.); nwi@nordicbio.com (N.W.); 2 Department of Biomedical Sciences, University of Copenhagen, 2200 Copenhagen, Denmark; 3 Department of Hematology, Zealand University Hospital, 4000 Roskilde, Denmark; mokl@regionsjaelland.dk (M.K.L.); vihs@regionsjaelland.dk (V.S.); laskj@regionsjaelland.dk (L.K.); trak@regionsjaelland.dk (T.A.K.); hkhl@regionsjaelland.dk (H.C.H.); 4 Department of Rheumatology, Odense University Hospital, 5000 Odense, Denmark; peter.junker@rsyd.dk

**Keywords:** myeloproliferative neoplasms (MPNs), circulating extracellular matrix (ECM) proteins, neoepitopes, biomarkers, type I collagen, type III collagen, type IV collagen, JAK2 variant allele frequency, bone marrow fibrosis, inflammation

## Abstract

**Background and objectives**: Myeloproliferative neoplasms (MPNs) are blood cancers characterized by elevated blood cell counts, bone marrow fibrosis (BMF), and chronic inflammation, which drives disease progression. BMF results from disrupted collagen turnover in the bone marrow extracellular matrix (ECM), making its reduction or stabilization a key therapeutic goal. Non-invasive biomarkers reflecting collagen turnover could potentially improve early detection of BMF and monitor disease activity. **Methods:** We evaluated serum biomarkers of collagen turnover in 130 MPN patients (MPN subtypes: ET = 49, PV = 60, pre-PMF = 8, PMF = 13) included in the DALIAH trial (ClinicalTrials.gov identifier: #NCT01387763). Type I and type III collagen formation (PRO-C1 and PRO-C3) and MMP-degraded type I, III, and IV collagens (C1M, C3M, and C4M) were measured by ELISA in serum. Biomarker levels were compared to age- and sex-matched healthy individuals and were assessed according to disease subtypes, somatic driver mutations, *JAK2V617F* VAF, and fibrosis grade. Furthermore, we studied correlations between the biomarker levels and conventional hematological markers for disease activity, such as hemoglobin, white blood cell count (WBCs), platelet counts, and lactate dehydrogenase (LDH). **Results:** Baseline PRO-C3 (*p* = 0.0005) and C1M (*p* = 0.0102) were elevated in MPN patients compared to healthy individuals, whereas C3M was decreased (*p* < 0.0001). Patients with primary myelofibrosis (PMF) had higher levels of PRO-C3 compared to patients with ET (*p* = 0.0041) and PV (*p* = 0.0172), correlated with higher *JAK2V617F* VAF (≥50%) (*p* = 0.0069) and advanced fibrosis grade (*p* = 0.0002). In addition, PRO-C3 was positively correlated to LDH, which is a biomarker of disease activity in MPNs (r = 0.5754, *p* < 0.0001). **Conclusions:** Taken together, these findings emphasize the role of ECM remodeling in MPN pathophysiology and the potential of soluble ECM neoepitopes as biologically plausible disease markers.

## Simple Summary

Myeloproliferative neoplasms (MPNs) are blood cancers associated with increased blood cell production, chronic inflammation, and bone marrow fibrosis. As fibrosis is linked to changes in collagen turnover in the bone marrow, blood-based biomarkers of collagen formation and degradation may help detect and monitor the disease. This study investigated several serum biomarkers of collagen turnover in patients with different MPN subtypes and examined how they relate to *JAK2V617F* VAF, fibrosis severity, and standard markers of disease activity.

## 1. Introduction

The classical chronic Philadelphia-negative myeloproliferative neoplasms (MPNs) include essential thrombocythemia (ET), polycythemia vera (PV), prefibrotic myelofibrosis (pre-MF), and primary myelofibrosis (PMF) [1,2,3]. They are clonal hematopoietic stem cell disorders characterized by elevated blood cell counts and develop over decades in a biological continuum from the earliest MPN stages (ET and PV) to the terminal MF stage, and in a subset thereafter leukemic transformation. In the advanced stage of PMF and MF following ET and PV, post-ET and post-PV, respectively, cytopenias are prevailing in terms of anemia and thrombocytopenia, being explained by bone marrow failure due to qualitatively defective hematopoiesis, progressive accumulation of connective tissue in the bone marrow, and splenic pooling and sequestration of blood cells in the enlarged spleen [4]. MPNs arise due to acquired somatic driver mutations, including *JAK2V617F*, *CALR* (the gene encoding calreticulin), or *MPL* (the thrombopoietin receptor gene) [5,6]. In most *JAK2V617F*-negative ET and PMF patients, *CALR* or *MPL* mutations are detectable, leaving about 10% of patients with MPN carrying none of the three driver mutations. These patients are termed triple-negative MPNs [7,8]. The *JAK2V617F* mutation is the most prevalent, occurring in nearly all patients with PV (98%) and in approximately 50–60% of patients diagnosed with ET or PMF.

Today, it is widely recognized that MPNs are driven by chronic inflammation [9]. Chronic inflammation may ultimately lead to bone marrow failure in MPN patients through changes in the bone marrow stroma and the progressive accumulation of fibrous tissue [10,11]. Fibrosis is the consequence of excessive deposition of extracellular matrix (ECM) components, such as collagens. In healthy tissue, a balanced ratio between ECM degradation and formation maintains tissue structure and functions, whereas chronic inflammation with resulting altered turnover of type I collagen and type III collagen (linked to ‘collagen’ and ‘reticulin’ fibrosis, respectively) in the ECM leads to bone marrow fibrosis (BMF) [12,13]. Altered collagen turnover implies enhanced neoformation and increased cleavage of collagens by proteases, e.g., matrix metalloproteinases (MMPs). These resulting fragments contain specific neo-epitopes, which act as ‘protein fingerprints’. They are released into the extracellular space and enter the bloodstream and can serve as serological biomarkers that reflect current collagen formation and degradative activities [14]. Since BMF is the final outcome, there is an urgent need to identify and evaluate novel non-invasive biomarkers of bone marrow fibrogenesis and angiogenesis, which develop in MPNs due to inflammation-driven progressive clonal expansion and evolution.

In the 1980s, studies showed the potential of circulating ECM fragments released to the circulation as markers of early bone marrow inflammation and late fibrogenesis, reflecting disease activity [15,16,17]. In recent years, the research field of utilizing circulating ECM fragments as biomarkers assessed by novel technologies has expanded immensely in many diseases, including cancer [18,19]. All fibrillar collagens, such as collagen types I and III, are synthesized as procollagens, which contain additional pro-peptide extensions at both ends. In the process of collagen fiber formation, the pro-peptide regions are cleaved off in the extracellular space and released into the bloodstream. This is utilized in newly developed, non-invasive biomarker assays that reflect the formation or degradation of various ECM proteins such as collagens. These biomarkers may have the potential to enhance early detection of BMF and disease activity in patients with MPNs, as well as to monitor the treatment effect [20]. In serum samples, PRO-C3 and PRO-C1 biomarkers detect protein fragments with fingerprints of type III collagen formation and type I collagen formation, respectively [21,22,23]. Specific MMP-generated protein fragments derived from type I, III, and IV collagen can be measured using the protein fingerprint biomarkers C1M, C3M, and C4M, respectively. They reflect degradation of the interstitial matrix (C1M and C3M) and basement membrane (C4M) [24,25,26].

In this study, we investigated the potential of the non-invasive biomarkers PRO-C3, PRO-C1, C3M, C1M, and C4M to assess disease activity, bone marrow fibrosis, and angiogenesis using pre-treatment serum samples from a large cohort of patients diagnosed with MPNs at different stages of their development using the DALIAH trial.

## 2. Materials and Methods

### 2.1. Patients and Controls

The DALIAH trial was an investigator-initiated, randomized, controlled, phase 3 clinical trial (ClinicalTrials.gov identifier: #NCT01387763) conducted at 8 study sites in Denmark. Enrollment began in February 2012 and was completed in July 2015. In this trial, the safety and efficacy of different pegylated IFN-α formulations (IFNα-2a and IFNα-2b) were compared with HU treatment in newly diagnosed MPN patients. Patients aged ≥ 18 years with a diagnosis of ET, PV, pre-MF, or PMF according to the World Health Organization (WHO) 2008 criteria were eligible for enrollment [2,27]. The DALIAH trial was approved by the Danish Medicines Agency and was conducted in compliance with the Declaration of Helsinki and Good Clinical Practice. Patients aged >60 years were randomly allocated (1:1:1) to receive HU, IFNα-2a, or IFNα-2b, whereas patients aged ≤60 years were randomly allocated (1:1) to receive IFNα-2a or IFNα-2b. The treatment dose was modified based on efficacy and toxicity according to predefined dose levels described elsewhere [28].

The present study includes pre-treatment serum (i.e., baseline) from 130 patients enrolled in the DALIAH trial. Before blood sampling for biomarker analysis and before randomization to HU, IFNα-2a or IFNα-2b, 15 out of 130 patients had shortly initiated treatment with HU due to “high-risk-profile” (major thrombosis at the time of diagnosis). Control serum samples from healthy individuals were obtained from the commercial supplier BioIVT (Westbury, NY, USA). BioIVT (Westbury, NY, USA) operates under the approval of an appropriate institutional review board/independent ethical committee for sample collection. All investigations were carried out in accordance with the Helsinki Declaration.

All samples were processed according to standardized operating procedures and stored at −80 °C until analysis. Furthermore, all samples underwent a maximum of 4 freeze–thaw cycle(s) prior to ELISA measurements in accordance with the individual assay validation data that supports analyte recovery for up to 5 freeze–thaw cycles.

### 2.2. Biomarker Measurements by ELISA

Levels of ECM biomarkers (PRO-C3, C3M, PRO-C1, C1M, and C4M) were quantified in serum using well-characterized competitive enzyme-linked immunosorbent assays (ELISAs) manufactured by Nordic Bioscience (Herlev, Denmark) and performed according to the manufacturer’s specifications. Each ECM fragment has a specific protease-generated neo-epitope that the monoclonal antibodies used in the ELISAs are highly specific against. PRO-C3 is generated by the N-protease-mediated release of the N-terminal pro-peptide of type III collagen, C3M is from inflammation-driven matrix metalloproteinase (MMP)-degraded type III collagen, PRO-C1 is generated by the N-protease-mediated release of the N-terminal pro-peptide of type I collagen, C1M is from inflammation-driven MMP-degraded type I collagen, and C4M measures MMP-degraded type IV collagen, as described in detail in Table 1. All serum samples were measured in duplicate.

### 2.3. JAK2V617F VAF and Fibrosis Grading

The *JAK2V617F* VAF was measured using allele-specific quantitative polymerase chain reaction (qPCR) on peripheral blood (assay sensitivity: 0.1%), described in detail by Larsen et al. [29].

The fibrosis grading was made according to the European Consensus grading of bone marrow fibrosis going from normal bone marrow with scattered linear reticulin fibers with no cross-over (i.e., grade 0) to grade 3 with a diffuse and dense increase in reticulin fibers with extensive cross-overs [30].

### 2.4. Statistical Analyses

Data were log-transformed prior to analysis to improve normality and reduce heteroscedasticity. Data are presented as original values on a logarithmic scale. Biomarker levels in patients with MPNs were compared to age- and sex-matched healthy individuals by using unpaired *t*-tests. In addition, the biomarker levels were evaluated according to disease subtypes, somatic driver mutation status, *JAK2V617F* VAF, and fibrosis grade, employing ordinary one-way ANOVA for statistical analysis. Non-invasive biomarker levels were correlated to conventional hematological biomarkers for disease activity in MPNs using Pearson’s correlation. Statistical analyses were performed using GraphPad Prism version 10.2.3 (GraphPad Software, San Diego, CA, USA). A *p*-value < 0.05 was considered statistically significant.

## 3. Results

### 3.1. Baseline Characteristics of MPN Patients

Baseline serum samples from 130 patients were included and measured by ELISAs to evaluate the levels of the ECM biomarkers PRO-C3, C3M, PRO-C1, C1M, and C4M. Fifteen of 130 patients with MPNs received short-term HU treatment before study initiation. As these patients were included in the baseline analyses, biomarker levels were compared between treatment-naïve patients and those pretreated with HU, revealing no differences between the groups (data shown in Supplementary Materials, Figure S1). Baseline characteristics of MPN patients and healthy individuals are shown in Table 2.

### 3.2. Collagen Biomarkers Are Altered in Patients with MPNs Compared to Healthy Individuals

**Figure 1:**
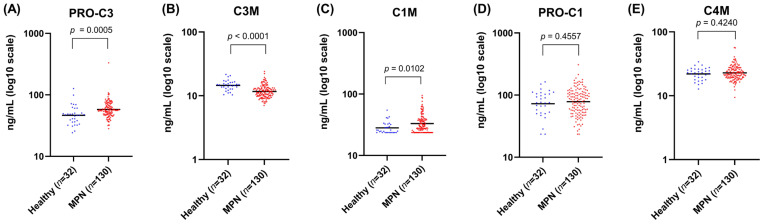
Serum PRO-C3 (**A**), C3M (**B**), C1M (**C**), PRO-C1 (**D**), and C4M (**E**) levels in patients with MPNs (*n* = 130) compared to healthy individuals (*n* = 32). Data points are plotted with geometric mean. Statistical differences were analyzed using an unpaired *t*-test.

When evaluating baseline biomarker levels in MPN patients compared to healthy individuals, PRO-C3 (*p* = 0.0005, 95% CI = 0.043, 0.148) and C1M (*p* = 0.0102, 95% CI = 0.017, 0.125) were significantly elevated in MPN patients as compared to healthy individuals (Figure 1A,C). In contrast, C3M was significantly decreased in patients with MPNs (*p* < 0.0001, 95% CI = −0.132, −0.059) (Figure 1B). PRO-C1 and C4M did not show any significant difference between patients with MPNs and healthy individuals (*p* = 0.4557, 95% CI = −0.053, 0.117, and *p* = 0.4240, 95% CI = −0.026, 0.060, respectively) (Figure 1D,E).

**Figure 2:**
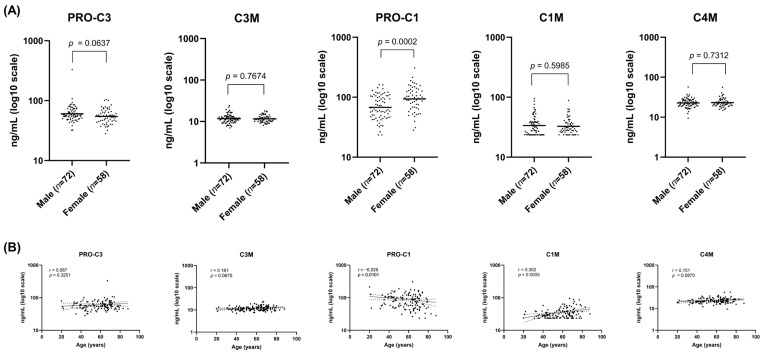
Sex and age correlation for MPN patients at baseline. Baseline serum levels of PRO-C3, C3M, PRO-C1, C1M, and C4M separated in sex (**A**). Individual data points are plotted with geometric mean. Statistical differences were analyzed using an unpaired *t*-test. Biomarker serum levels correlated with the age of the patients at baseline (**B**). All data points are plotted individually from the patients. The solid line shows simple linear regression of the correlation with 95% CI as dashed lines. Statistical correlations were analyzed using Pearson’s correlation.

To evaluate whether these differences were associated with age or sex, we grouped patients accordingly to compare biomarker levels. When grouped by sex, no difference was seen for PRO-C3, C3M, C1M, and C4M, whereas PRO-C1 was significantly higher in women compared to men (*p* = 0.0002, 95% CI = 0.069, 0.213) (Figure 2A). When correlating to age, no correlation was found for PRO-C3, C3M and C4M; however, PRO-C1 (r = −0.225, *p* = 0.0101, 95% CI = −0.382, −0.055) and C1M (r = 0.302, *p* = 0.0005, 95% CI = 0.136, 0.451) were modestly correlated with age (Figure 2B).

Taken together, these findings indicate that collagen turnover in the ECM is altered in MPN patients compared to healthy individuals. Also, this alteration in MPN patients was found to depend on both sex and age for type I collagen biomarkers.

### 3.3. Associations Between Collagen Biomarkers and Conventional Biomarkers of Disease Activity

We performed association analyses between disease activity and levels of the serum biomarkers, using the conventional biomarkers of disease activity in MPNs (i.e., hemoglobin, hematocrit, white blood cell count (WBCs), neutrophil count, platelet count, and lactate dehydrogenase (LDH)).

**Figure 3:**
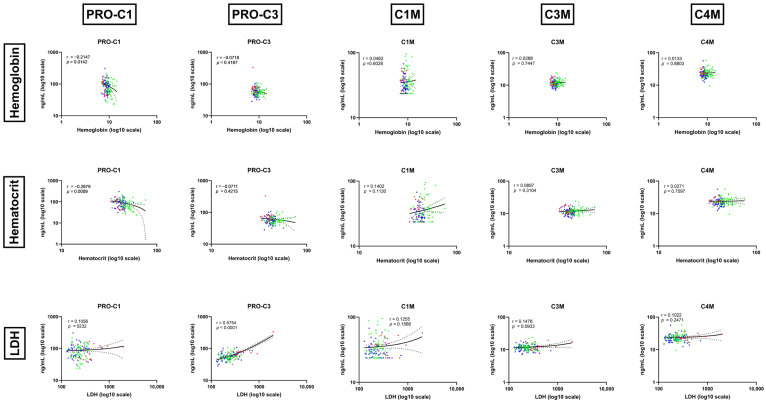
Correlation of non-invasive biomarker levels and conventional hematological biomarkers (hemoglobin (mmol/L), hematocrit (vol%) and LDH (U/L)) for disease activity in MPNs. All data points are plotted individually for each patient and divided into MPN subtypes (ET = blue, PV = green, pre-MF = purple, and PMF = red). Linear regression of the correlation is added as a solid line with 95% CI of the best-fitted line in dashed lines. Statistical correlations were analyzed using Pearson’s correlation.

We found that lower levels of PRO-C1 were associated with higher hemoglobin and hematocrit levels driven by the pre-MF patients (r = −0.2147, *p* = 0.0142, 95% CI = −0.373, −0.044 and r = −0.2879, *p* = 0.0009, 95% CI = −0.438, −0.122, respectively). Patients with higher LDH were found to have higher levels of PRO-C3 (r = 0.5754, *p* < 0.0001, 95% CI = 0.448, 0.680) (Figure 3).

**Figure 4:**
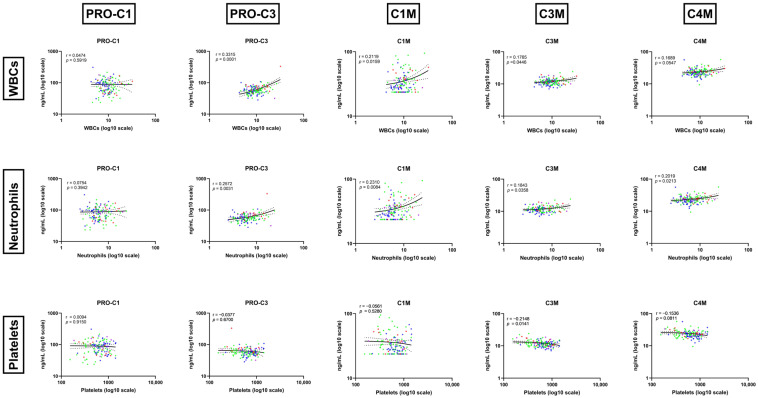
Correlation of non-invasive biomarker levels and conventional hematological blood cell counts (WBCs (×10^9^/L), neutrophils (×10^9^/L), and platelets (×10^9^/L)) in MPNs. All data points are plotted individually for each patient and divided into MPN subtypes (ET = blue, PV = green, pre-MF = purple, and PMF = red). Linear regression of the correlation is added as a solid line with 95% CI of the best-fitted line in dashed lines. Statistical correlations were analyzed using Pearson’s correlation.

In addition, higher levels of C3M correlate with lower platelet counts (r = −0.2148, *p* = 0.0141, 95% CI = −0.373, −0.044), particularly in PMF patients (r = −0.6559, *p* = 0.0149, 95% CI = −0.887, −0.164). Furthermore, higher levels of PRO-C3, C1M, C3M, and C4M correlated with higher levels of WBCs (r = 0.3315, *p* = 0.0001, 95% CI = 0.169, 0.477; r = 0.2119, *p* = 0.0159, 95% CI = 0.041, 0.371; r = 0.1765, *p* = 0.0446, 95% CI = 0.004, 0.338; and r = 0.1689, *p* = 0.0547, 95% CI = −0.003, 0.332, respectively). In regard to C3M and C4M, this association was strongest in patients with PV (r = 0.2563, *p* = 0.0481, 95% CI = 0.003, 0.479 and r = 0.2904, *p* = 0.0244, 95% CI = 0.039, 0.507, respectively). Also, the neutrophil count was positively correlated with higher levels of PRO-C3, C1M, C3M, and C4M (r = 0.2572, *p* = 0.0031, 95% CI = 0.089, 0.411; r = 0.2310, *p* = 0.0084, 95% CI = 0.061, 0.388; r = 0.1843, *p* = 0.0358, 95% CI = 0.013, 0.346; and r = 0.2019, *p* = 0.0213, 95% CI = 0.031, 0.361, respectively) (Figure 4). All individual Pearson correlation coefficients (r) and *p*-values can be seen in Appendix A Table A1.

### 3.4. Collagen Biomarkers Differ Between MPN Subtypes and Associate with the JAK2V617F VAF

**Figure 5:**
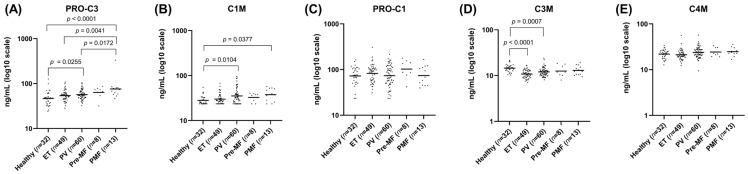
Serum PRO-C3 (**A**), C1M (**B**), PRO-C1 (**C**), C3M (**D**), and C4M (**E**) levels stratified by MPN subtypes (ET, PV, pre-MF, and PMF). Individual data points are plotted with geometric mean. Statistical differences were analyzed using one-way ANOVA.

Separating patients into the MPN subtypes (ET, PV, pre-MF, and PMF), PRO-C3 was significantly elevated in patients with PMF compared to patients with ET (*p* = 0.0041, 95% CI = −0.261, 0.086) and PV (*p* = 0.0172, 95% CI = −0.238, −0.015), as shown in Figure 5A. Neither C3M, PRO-C1, C1M nor C4M showed any differences in biomarker levels between MPN subtypes (Figure 5B–E).

**Figure 6:**
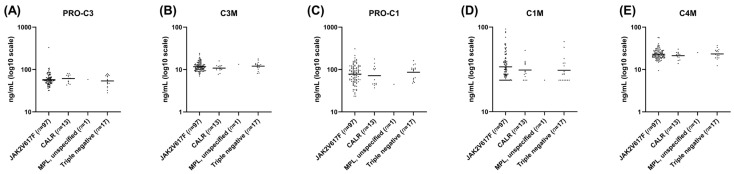
Serum PRO-C3 (**A**), C3M (**B**), PRO-C1 (**C**), C1M (**D**), and C4M (**E**) levels divided into the main driver mutations in MPNs (JAK2V617F, CALR, MPL, or triple negative). Individual data points are plotted with geometric mean. Statistical differences were analyzed using one-way ANOVA. No statistical differences were observed.

**Figure 7:**
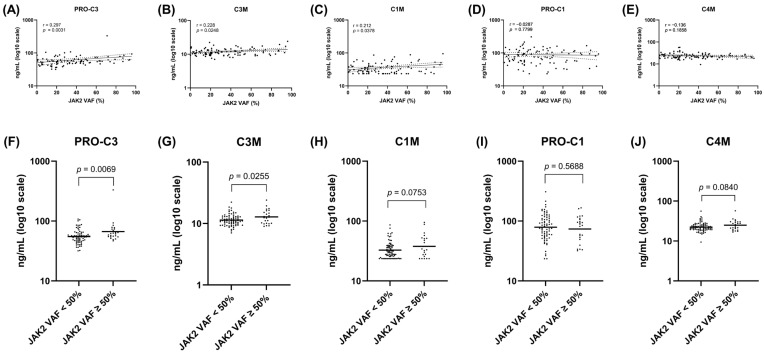
Biomarker serum levels correlated to the JAK2V617F VAF of the patients at baseline PRO-C3 (**A**), C3M (**B**), C1M (**C**), PRO-C1 (**D**), and C4M (**E**). All data points are plotted individually from the patients. The solid line shows simple linear regression of the correlation with 95% CI as dashed lines. Statistical correlations were analyzed using Pearson’s correlation. Pre-treatment serum PRO-C3 (**F**), C3M (**G**), PRO-C1 (**H**), C1M (**I**), and C4M (**J**) levels from patients with a JAK2V617F VAF <50% or ≥50% (*n* = 73 and *n* = 24, respectively). Individual data points are plotted with geometric mean, and statistical differences were analyzed using an unpaired *t*-test.

When evaluating biomarker levels according to somatic driver mutation status, no difference was observed in any of the collagen biomarker levels (Figure 6A–E). However, in the *JAK2V617F*-positive subgroup of MPN patients the levels of PRO-C3, C3M and C1M were found to associate with higher *JAK2V617F* VAF (r = 0.297, *p* = 0.0031, 95% CI = 0.104, 0.469; r = 0.228, *p* = 0.0248, 95% CI = 0.030, 0.409; and r = 0.212, *p* = 0.0378, 95% CI = 0.012, 0.396, respectively) (Figure 7A–C). Separating *JAK2V617F*-positive patients into high (≥50%) or low VAF (<50%), patients with high *JAK2V617F* VAF (≥50%) (*n*, PV = 19 and PMF = 5) had a significantly higher level of PRO-C3 (*p* = 0.0069, 95% CI = 0.023, 0.142) and C3M (*p* = 0.0255, 95% CI = 0.007, 0.098), as shown in Figure 7F,G.

Taken together these findings indicate that the type III collagen biomarkers were increased in MPN patients diagnosed with PMF compared to patients with ET or PV. Furthermore, the type III collagen biomarkers were in particular increased in patients with a *JAK2V617F* VAF of 50% or higher. These findings suggest that the type III collagen biomarkers can be biologically meaningful tools for the assessment of MPN disease activity and disease severity.

### 3.5. PRO-C3 Is Elevated in Patients with MPNs with Fibrosis Grade 2 + 3 Compared to Grade 0 and 1

**Figure 8:**
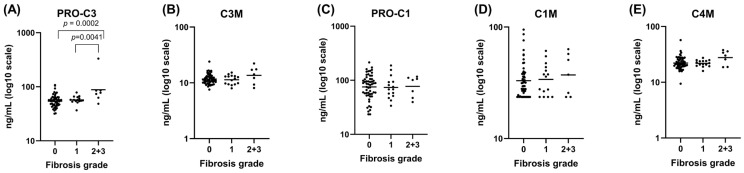
Serum PRO-C3 (**A**), C3M (**B**), PRO-C1 (**C**), C1M (**D**), and C4M (**E**) levels divided into the fibrosis stage (0: *n* = 56, 1: *n* = 15, 2 + 3: *n* = 7) at baseline in patients with MPNs. Individual data points are plotted with geometric mean. Statistical differences were analyzed using one-way ANOVA.

PRO-C3 at baseline was found to be elevated in patients with a fibrosis grade of 2 or 3 compared to patients with a fibrosis grade of 0 (*p* = 0.0002, 95% CI = −0.333, −0.093) or 1 (*p* = 0.0041, 95% CI = −0.326, −0.052), as shown in Figure 8A. Also, patients with PMF and *JAK2V617F* VAF > 50% were found to have a bone marrow fibrosis grade of 2 or 3. None of the other tested collagen biomarkers (C3M, PRO-C1, C1M, and C4M) were associated with fibrosis grade at baseline (Figure 8B–E).

Taken together, these findings indicate that the type III collagen biomarker PRO-C3 can serve as a bone marrow fibrosis marker in MPN patients.

## 4. Discussion

This study evaluated patients with MPNs at different disease stages from the DALIAH trial and healthy individuals, using serum biomarkers (PRO-C3, C3M, PRO-C1, C1M, and C4M) reflecting bone marrow stromal responses to clonal expansion.

Here we report that the biomarker of type III collagen synthesis (PRO-C3), a major constituent of the delicate reticulin fibrils, was increased, particularly in patients with PV and PMF, while its degradation (C3M) decreased. These findings correlated with conventional disease activity markers, bone marrow fibrosis grade and the *JAK2V617F* VAF. By contrast, C1M, reflecting ongoing degradation of type I collagen, which forms coarse mechanically stable fibers, was increased in MPN patients. PRO-C1, measuring type I collagen synthesis, and the basement membrane marker C4M, measuring type IV collagen metabolism, remained essentially inside the normal range.

The type III procollagen N-terminal pro-peptide (PIIINP) has been established as a reliable serological marker for type III procollagen synthesis in healthy people and an array of primary and secondary fibroproliferative conditions [15,16,17,31,32]. While the present results accord well with these earlier observations, they also add important novel information on circulating biomarkers of collagen metabolism in MPNs.

First, previous studies on the type III procollagen N-peptide were all based on various RIA technologies [15,32]. By contrast, the present data were obtained using a novel ELISA technology that detects the intact type III procollagen N-peptide (neo-epitope), which is cleaved off enzymatically from the parent procollagen molecule in a 1:1 ratio following secretion of the procollagen molecule into the extracellular space. This offers a unique opportunity to monitor the current type III collagen biosynthetic activity in blood by a marker molecule directly reflecting the fibroproliferative status related to the tissue response to injury, healing and regeneration instead of distant markers like CRP and LDH or previously deposited collagen in a bone marrow biopsy [21,33]. In regard to LDH, a well-known biomarker of tissue damage and disease activity in MPN patients, PRO-C3 was found to significantly correlate to a higher level of LDH. Previous studies showed that LDH was found to be elevated in patients with pre-MF and PMF [34], which correlates to the finding that PRO-C3 was associated with disease severity (*i.e*., MPN subtypes) and advanced fibrosis grade. Therefore, LDH and PRO-C3 go hand in hand and are both markers of disease activity, the important difference being that PRO-C3 directly reflects type III collagen synthesis in the bone marrow, whereas LDH mainly reflects the accelerated myeloproliferation only. As the highest levels of PRO-C3 were found in PMF patients (with reticulin fibrosis), this biomarker may have the potential to separate pre-MF from PMF patients more precisely than other biomarkers of disease activity, including LDH, taking into account pre-MF as defined by no or only slightly increased reticulin fibers (grade 1) in the bone marrow [35]. Even though pre-MF is WHO-defined and validated as a separate disease entity it entails certain concerns and limitations, which include the recognized difficulties amongst even well-trained hematopathologists to separate pre-MF from genuine ET and even from PV [35,36,37,38,39,40]. These still unsolved issues regarding its diagnostic reproducibility and its eligibility as a biological distinct disease entity call for novel technologies, such as measurements of circulating ECM proteins [41,42,43,44]. This might yield new insights on pre-MF as a separate MPN disease entity or if pre-MF is just aligning along a clinical and biological continuum featuring a mode of presentation of PMF with an indolent phenotype and a long survival during the patient’s journey from early stage MPN (ET, PV, Pre-MF) towards the advanced MF with bone marrow failure, huge splenomegaly and imminent leukemic transformation [45]. Since pre-MF also exhibited elevated C3M levels it is intriguing to speculate if no or slightly increased amounts of reticulin fibers in pre-MF reflect the increased degradation of type III collagen, which is equivalent to and matches the ongoing type III collagen synthesis.

Second, for the first time, we present data on the degradation of type III collagen (C3M) along with anabolic PRO-C3 results in MPNs, thereby providing an extended type III collagen metabolic profile. Surprisingly, we found that the significantly enhanced type III collagen synthetic rate (PRO-C3) thought to account for the increased bone marrow fibrosis grade, was accompanied by a marked decrease in the degradation of type III collagen (C3M), thereby strongly indicating that the MPN-related bone marrow fibrosis is driven by both pro-anabolic (e.g., platelet-derived growth factor (PDGF), transforming growth factor beta (TGF-β)) and anti-catabolic (e.g., inhibitors of collagen degradation—directly or indirectly) mediators.

Third, type III collagen is expressed early at sites of tissue injury irrespective of cause (physical, chemical, immunologic, etc.), forming a provisional argyrophilic fibril network serving as a template for the late-stage dense type I collagen fibrous deposits. Thus, it is of particular interest that not only PRO-C3 but also C3M and C1M correlated significantly with VAF in the *JAK2V617F*-positive MPN patients because this somatic mutation has, within recent years, been documented as an important proinflammatory and prothrombotic risk factor for, e.g., CVDs and thrombotic stroke, which are also prevalent in MPN patients [46,47]. This underscores the need for increasing awareness of ongoing inflammatory activities to gain early control over this important co-driver of bone marrow and systemic disease in the MPNs. Based on the present observations, PRO-C3 is a promising and biologically plausible blood-borne candidate marker for this clinical purpose. The observation of a tight association between *JAK2V617F* and several ECM proteins, including PRO-C3, C3M and C1M is even more intriguing when considering the work by Passamonti’s group in 2006 on the relation between *JAK2V617F* mutation, granulocyte activation and constitutive mobilization of CD34+ cells into peripheral blood in MPNs and their later highly important paper on the association between the *JAK2V617F* VAF and transformation to myelofibrosis in MPNs [48,49]. Our findings align perfectly with those of Passamonti et al., and together they supplement and enrich each other by building a pathophysiological model for the interpretation of our results. This model connects *JAK2V617F* to dual, opposing processes in the bone marrow—fibrogenesis (via megakaryocyte/monocyte production of GF mitogenic for fibrogenesis and angiogenesis) and proteolysis (via granulocyte MMP release). The model accurately describes the dynamic ECM remodeling in MPNs in terms of several important aspects. First, it addresses the key topic regarding the *JAK2V617F*, clonal fitness and fibrosis risk. In this regard the tight association between a high *JAK2V617F* VAF above 50% and C3M is crucial, considering that a *JAK2V617F* VAF above 50% is also a specific risk factor for progression to myelofibrosis in PV [49]. In this regard, our findings support the concept that the biological activity of the *JAK2V617F* mutation, not just its presence, dictates the fibrotic microenvironment. Second, the model also fits with the “Tipping Point” and MPN fitness—a measure of how effectively the mutant clone outcompetes normal hematopoiesis across different cell lineages, in which the balance between collagen synthesis and degradation may be central. Shifts in the ECM biomarkers (like PRO-C3 and C1M) likely reflect changes in this underlying clonal fitness, helping to explain why the balance tips toward net fibrosis in some patients. This fitness has been shown to be a more powerful predictor of clinical outcome than the simple whole-blood allele frequency [50]. Given the tight association between C3M and the *JAK2V617F* VAF, it is intriguing to speculate if a simple ‘liquid biopsy’ of the bone marrow by measuring a few ‘key’ ECM proteins, including PRO-C3, C3M, C1M and their ratios as likely the most robust, might eventually indicate highly important biomarkers of bone marrow fibrogenic activity, serve as predictors of prognosis and allow the monitoring of responses to treatment. However, this needs to be further explored in future studies [51,52].

Type I collagen is the most abundant structural protein in human tissues. It provides mechanical strength and resilience and co-regulates growth, cell proliferation, and differentiation, including the basic inflammatory response to injury which terminates with either repair and healing or continues as a default process leading to fibrosis, rich in type I collagen coarse fibers [53]. While the type III collagen serological profile was clearly anabolic (increased PRO-C3 and decreased C3M), the pattern for type I collagen was less clear. Here, we observed that the PRO-C1 marker which measures the N-terminal peptide of type I collagen was largely within the normal range. However, we expected from previous findings to see an increase in PRO-C1 in more advanced disease stages, as collagen fibrosis (i.e., more type I collagen in the bone marrow) has been associated with abnormal blood counts and severity of underlying disease [54,55]. One take on this could be that the rate of new type I collagen synthesis may not be dramatically increased, even as collagen accumulates. Conversely, the degradation marker, C1M correlated with the platelet count in the ET subset, suggesting a regulatory link at this early MPN disease stage between platelet activation and increased platelet egress from the BM compartment to the systemic circulation, likely associated with release of proteolytic enzymes capable of degrading type I collagen fragments initially generated by local collagenase activity. A similar pattern has previously been reported in patients with PV and MF [31]. The elevation of C1M is a direct sign of active ECM proteolysis in the bone marrow microenvironment, in which chronic inflammation, driven by cytokines and activated granulocytes and monocytes leads to sustained release of MMPs (e.g., MMP-2, -9, -13). This increases collagen breakdown, releasing C1M fragments into the circulation. The finding of elevated C1M even in a subset of patients with early stage MPN (ET) is particularly astonishing. It suggests that ongoing inflammatory tissue degradation and remodeling begin early in the disease course, preceding visible fibrosis on biopsy. This could represent a “high-turnover” state where both degradation, and to a lesser extent, synthesis are heightened.

The collagen biomarker C4M reflects type IV collagen degradation and thereby basement membrane remodeling, which is associated with malignant progression and metastatic dissemination [56]. However, in this cohort of patients, C4M did not show any significant differences in baseline characteristics. It has previously been shown that type IV collagen was increased (by a serum marker measuring the 7S domain of type IV collagen) in PMF patients compared to healthy individuals [16]. This could indicate that the increase in MPN patients might come from increased formation of type IV collagen and not from a decreased degradation of type IV collagen (measured by C4M), as we did not observe that from this study.

This study carries some limitations. One major limitation of this study is the limited group sizes and the varying number of patients in each group. One example is the subgrouping by driver mutations, where higher numbers of patients are in the group of *JAK2V617F*-positive patients as compared to *MPL* patients. Also, a few patients were excluded due to insufficient description of the degree of bone marrow fibrosis, or the biopsy was not suitable due to poor quality. Another limitation is that the diagnoses were assigned locally according to the contemporaneous WHO 2008 criteria [2]. In these criteria pre-MF was not yet established as a separate disease entity, but the classification did include the concept of a pre-fibrotic cellular phase of PMF based on characteristic megakaryocytic morphology and bone marrow cellularity features, used for establishing the pre-MF group in this study. Re-classification according to WHO 2022 criteria [57], including pre-MF as a separate disease entity, was not done for this study due to original biopsy material not being systematically available. Finally, the healthy control group is also of limited size and the age discrepancy between the healthy control group and the MPN cohort represents an important limitation. Therefore, the inclusion of an age-matched control group would be critical for more robust conclusions. However, we believe that our control group is appropriate considering that no differences in biomarker levels were recorded between individuals with an age below 50 years and above 50 years, respectively (data shown in Supplementary Materials, Figure S2). Furthermore, previous independent studies on the relationship between the type III N-terminal pro-peptide of type III collagen (PIIINP) and age using different assay platforms have yielded small and non-systematic decade-related deviations between subjects in different age groups suggesting that partitioning according to age within the reference age is not needed [58,59]. The small subgroups in this cohort possibly diminish the statistical power.

## 5. Conclusions

In MPN patients, type I and type III collagen turnover are altered, likely reflecting increased disease activity. The non-invasive fibrosis-associated biomarker PRO-C3 was found to be elevated in patients with PMF compared to ET or PV and in patients with a *JAK2V617F* allele burden ≥ 50% indicating that PRO-C3 may be a marker of MPN disease severity. In further support of this, PRO-C3 also correlated with fibrosis grade in the bone marrow of MPN patients, with patients with a more severe fibrosis stage showing higher PRO-C3 levels. Therefore, PRO-C3 and other serological collagen biomarkers show potential as valuable non-invasive bone marrow fibrosis biomarkers, useful for stratifying and monitoring disease progression in patients with MPNs.

## Figures and Tables

**Table 1:** Biomarker overview.

Biomarker Name	Description	Target Site	Purpose
PRO-C1	Type I collagen formation	N-terminal pro-peptide of type I collagen	A marker of type I collagen formation
PRO-C3	Type III collagen formation	N-terminal pro-peptide of type III collagen	A marker of fibroblast activity (fibrogenesis)
C1M	Inflammation-driven MMP-degraded type I collagen	MMP-cleavage site	A marker of type I collagen degradation
C3M	Inflammation-driven MMP-degraded type III collagen	MMP-cleavage site	A marker of interstitial type III collagen matrix degradation and remodeling
C4M	MMP-degraded type IV collagen	MMP-cleavage site	A marker of basement membrane degradation and remodeling

**Table 2:** Baseline characteristics of all patients and those who have been treated shortly with HU before randomization due to “high-risk-profile” as well as healthy individuals. Numbers are median (range), or *n* (%).

Patient Characteristics	Total MPN Patients (*n* = 130) (%)	HU Treatment Before Study Start (*n* = 15) (%)	Healthy Individuals (*n* = 32) (%)
Biological sex			
Male	72 (56)	10 (67)	16 (50)
Female	58 (44)	5 (33)	16 (50)
Age (years)	61 (20–88)	64 (33–88)	47 (19–72)
Age male	62 (31–83)	60 (33–78)	53 (29–72)
Age female	57 (20–88)	65 (63–88)	39 (19–64)
MPN subtype			
ET	49 (38)	3 (20)	-
PV	60 (47)	9 (60)	-
Pre-MF	8 (6)	2 (13)	-
PMF	13 (9)	1 (7)	-
Somatic driver mutation			
*JAK2V617F*	97 (75)	12 (80)	-
*CALR*	13 (10)	2 (13)	-
*MPL*	1 (1)	0 (0)	-
Triple negative	17 (14)	1 (7)	-
*JAK2V617F* VAF (%)	28 (1–96)	45 (15–79)	-
Bone marrow fibrosis grade *			
0	56 (72)	5 (56)	-
1	15 (19)	2 (22)	-
2	6 (8)	2 (22)	-
3	1 (1)	0 (0)	-
Hematological variables			
Hemoglobin (g/L)	9 (7–14.5)	9 (7.3–14.5)	-
Hematocrit (vol %)	44 (33–68)	43 (33–68)	-
WBCs (×10^9^/L)	9.4 (4.6–32.4)	9.2 (4.7–32.4)	-
Platelets (×10^9^/L)	643 (154–1441)	662 (154–1150)	-
LDH (U/L)	241 (129–1995)	293 (136–1995)	-
Neutrophil (×10^9^/L)	6.2 (2.5–24.2)	6.1 (3.1–16.8)	-

* Note only 78/130 patients at baseline had a measured bone marrow fibrosis grade.

## Data Availability

The materials and data used in this study are available upon reasonable request. Some restrictions may apply due to Material Transfer Agreements (MTAs) or institutional policies, which are necessary to protect proprietary information or intellectual property rights. Requests for materials should be directed to the corresponding author, Caroline Norup Bistrup, cabi@nordicbio.com.

## Supplementary Materials

The following supporting information can be downloaded at: https://www.mdpi.com/article/10.3390/cancers18152529/s1, Figure S1: Serum PRO-C3 (**A**), C3M (**B**), PRO-C1 (**C**), C1M (**D**), and C4M (**E**) levels in patients with MPNs (n = 130) divided into treatment-naïve patients (n = 115) and patients that received HU before study start (n = 15). Data points are plotted with geometric mean. Statistical differences were analyzed using an unpaired *t*-test. Figure S2: Serum PRO-C3 (**A**), PRO-C1 (**B**), C3M (**C**), C1M (**D**), and C4M (**E**) levels in healthy individuals (*n* = 32) divided into two age groups—one group consisting of individuals with an age below 50 years (*n* = 18, average age = 34 years) and one group with an age of more than 50 years (*n* = 14, average age = 59 years). Data points are plotted with geometric mean. Statistical differences were analyzed using an unpaired *t*-test.

## Abbreviations

The following abbreviations are used in this manuscript:

MPN	Myeloproliferative neoplasms
VAF	Variant allele frequency
BMF	Bone marrow fibrosis
ECM	Extracellular matrix
ET	Essential thrombocythemia
PV	Polycythemia vera
Pre-MF	Prefibrotic myelofibrosis
PMF	Primary myelofibrosis
*CALR*	The gene encoding calreticulin
*MPL*	The thrombopoietin receptor gene
MMP	Matrix metalloproteinase
WHO	World Health Organization
IFNα-2	Interferon alpha 2
HU	Hydroxyurea
ELISA	Enzyme-linked immunosorbent assay
qPCR	Quantitative polymerase chain reaction
LDH	Lactate dehydrogenase
CRP	C-reactive protein
PDGF	Platelet-derived growth factor
TGF-β	Transforming growth factor beta
WBCs	White blood cell

## Appendix A

**Table A1:** Correlations of ECM biomarker levels with conventional hematological markers of MPN disease. r is the Pearson correlation coefficient. Statistical significance is highlighted in bold.

Biomarker/MPN Subtype	Hemoglobin (mmol/L)	Hematocrit (vol %)	WBCs (×10^9^/L)	Platelets (×10^9^/L)	LDH (U/L)	Neutrophils (×10^9^/L)
**PRO-C1**						
All MPN patients	**r = −0.2147** **(*p* = 0.0142)**	**r = −0.2879** **(*p* = 0.0009)**	r = 0.0474 (*p* = 0.5919)	r = 0.0094 (*p* = 0.9150)	r = 0.1056 (*p* = 0.232)	r = 0.0754 (*p* = 0.3942)
ET	r = −0.0394 (*p* = 0.7881)	r = −0.0167 (*p* = 0.9094)	r = −0.1642 (*p* = 0.2596)	r = −0.0824 (*p* = 0.5735)	r = −0.0218 (*p* = 0.8817)	r = −0.0198 (*p* = 0.8925)
PV	r = −0.1902 (*p* = 0.1454)	**r = −0.3327** **(*p* = 0.0094)**	r = 0.1150 (*p* = 0.3816)	r = 0.0969 (*p* = 0.4616)	r = 0.0715 (*p* = 0.5870)	r = 0.1162 (*p* = 0.3764)
Pre-MF	**r = −0.8494** **(*p* = 0.0076)**	**r = −0.7718** **(*p* = 0.0249)**	r = −0.0909 (*p* = 0.8306)	r = −0.0202 (*p* = 0.9621)	r = 0.3881 (*p* = 0.3421)	r = −0.06342 (*p* = 0.8814)
PMF	**r = −0.5733** **(*p* = 0.0405)**	r = −0.4790 (*p* = 0.0977)	r = 0.3550 (*p* = 0.2339)	r = −0.5044 (*p* = 0.0788)	r = 0.4588 (*p* = 0.1148)	r = 0.4065 (*p* = 0.1680)
**PRO-C3**						
All MPN patients	r = −0.0718 (*p* = 0.4167)	r = −0.0711 (*p* = 0.4215)	**r = 0.3315** **(*p* = 0.0001)**	r = −0.0377 (*p* = 0.6700)	**r = 0.5754** **(*p* < 0.0001)**	**r = 0.2572** **(*p* = 0.0031)**
ET	r = 0.0372 (*p* = 0.7998)	r = −0.0986 (*p* = 0.5003)	r = −0.2252 (*p* = 0.1197)	**r = 0.3877** **(*p* = 0.0059)**	**r = 0.3366** **(*p* = 0.0181)**	r = −0.1191 (*p* = 0.4148)
PV	r = 0.0762 (*p* = 0.5626)	r = 0.1260 (*p* = 0.3376)	**r = 0.4523** **(*p* = 0.0003)**	r = −0.1053 (*p* = 0.4234)	r = 0.1871 (*p* = 0.1522)	**r = 0.4314** **(*p* = 0.0006)**
Pre-MF	r = −0.3601 (*p* = 0.3809)	r = −0.3448 (*p* = 0.4029)	**r = −0.8445** **(*p* = 0.0083)**	r = −0.2171 (*p* = 0.6055)	r = 0.0814 (*p* = 0.8480)	**r = −0.8264** **(*p* = 0.0114)**
PMF	r = −0.1649 (*p* = 0.5904)	r = −0.1783 (*p* = 0.5601)	**r = 0.7274** **(*p* = 0.0048)**	r = 0.4931 (*p* = 0.0869)	**r = 0.8983** **(*p* < 0.0001)**	r = 0.4924 (*p* = 0.0873)
**C1M**						
All MPN patients	r = 0.0462 (*p* = 0.6028)	r = 0.1402 (*p* = 0.1130)	**r = 0.2119** **(*p* = 0.0159)**	r = −0.0561 (*p* = 0.5280)	r = 0.1255 (*p* = 0.1566)	**r = 0.2310** **(*p* = 0.0084)**
ET	r = −0.1329 (*p* = 0.3627)	r = −0.1607 (*p* = 0.2699)	r = 0.0423 (*p* = 0.7727)	**r = 0.4234** **(*p* = 0.0024)**	r = −0.1071 (*p* = 0.4637)	r = 0.0657 (*p* = 0.6540)
PV	r = 0.0233 (*p* = 0.8612)	r = 0.1625 (*p* = 0.2188)	r = 0.2228 (*p* = 0.0899)	r = −0.1061 (*p* = 0.4240)	r = 0.0928 (*p* = 0.4845)	r = 0.2401 (*p* = 0.0670)
Pre-MF	r = 0.0818 (*p* = 0.8474)	r = 0.1627 (*p* = 0.7002)	r = −0.2889 (*p* = 0.4877)	r = −0.6358 (*p* = 0.0902)	**r = −0.7266** **(*p* = 0.0412)**	r = −0.2763 (*p* = 0.5077)
PMF	r = 0.1371 (*p* = 0.6551)	r = 0.2978 (*p* = 0.3231)	r = 0.1907 (*p* = 0.5327)	r = −0.2174 (*p* = 0.4756)	r = 0.3284 (*p* = 0.2733)	r = 0.1752 (*p* = 0.5671)
**C3M**						
All MPN patients	r = 0.0288 (*p* = 0.7447)	r = 0.0897 (*p* = 0.3104)	**r = 0.1765** **(*p* = 0.0446)**	**r = −0.2148** **(*p* = 0.0141)**	r = 0.1478 (*p* = 0.0933)	**r = 0.1843** **(*p* = 0.0358)**
ET	r = −0.0155 (*p* = 0.9159)	r = −0.0313 (*p* = 0.8307)	r = −0.0744 (*p* = 0.6117)	r = 0.0721 (*p* = 0.6226)	r = −0.1739 (*p* = 0.2320)	r = −0.0824 (*p* = 0.5738)
PV	r = 0.1130 (*p* = 0.3901)	r = 0.2226 (*p* = 0.0873)	**r = 0.2563** **(*p* = 0.0481)**	r = −0.1969 (*p* = 0.1315)	r = −0.0001 (*p* = 0.9991)	**r = 0.2901** **(*p* = 0.0245)**
Pre-MF	r = −0.4727 (*p* = 0.2369)	r = −0.4267 (*p* = 0.2917)	r = −0.3211 (*p* = 0.4380)	r = −0.3160 (*p* = 0.4458)	r = −0.4625 (*p* = 0.2486)	r = −0.2974 (*p* = 0.4744)
PMF	r = −0.4384 (*p* = 0.1340)	r = −0.3377 (*p* = 0.2591)	r = 0.1431 (*p* = 0.6410)	**r = −0.6559** **(*p* = 0.0149)**	r = 0.4580 (*p* = 0.1155)	r = −0.0114 (*p* = 0.9704)
**C4M**						
All MPN patients	r = 0.0133 (*p* = 0.8803)	r = 0.0271 (*p* = 0.7597)	r = 0.1689 (*p* = 0.0547)	r = −0.1536 (*p* = 0.0811)	r = 0.1022 (*p* = 0.2471)	**r = 0.2019** **(*p* = 0.0213)**
ET	r = −0.0920 (*p* = 0.5298)	r = −0.0991 (*p* = 0.4980)	r = −0.2568 (*p* = 0.0749)	r = −0.0421 (*p* = 0.7742)	r = −0.1195 (*p* = 0.4135)	r = −0.1996 (*p* = 0.1691)
PV	r = 0.0227 (*p* = 0.8633)	r = 0.0272 (*p* = 0.8366)	**r = 0.2904** **(*p* = 0.0244)**	r = −0.0600 (*p* = 0.6486)	r = 0.1083 (*p* = 0.4103)	**r = 0.3034** **(*p* = 0.0185)**
Pre-MF	r = −0.4107 (*p* = 0.3122)	r = −0.3339 (*p* = 0.4189)	r = −0.1482 (*p* = 0.7262)	r = −0.3464 (*p* = 0.4006)	r = −0.5027 (*p* = 0.2042)	r = −0.1093 (*p* = 0.7966)
PMF	r = −0.2176 (*p* = 0.4751)	r = −0.0575 (*p* = 0.8519)	r = 0.1874 (*p* = 0.5398)	r = −0.4688 (*p* = 0.1061)	r = 0.3584 (*p* = 0.2292)	r = 0.1351 (*p* = 0.6599)
