# Supplementary material for: Collagen Turnover Is Associated with Disease Severity, Bone Marrow Fibrosis, and the JAK2V617F Variant Allele Frequency in Myeloproliferative Neoplasms
Source: Cancers (Basel). 2026 Aug 6;18(15):2529. doi: 10.3390/cancers18152529 (PMC13465665; doi:10.3390/cancers18152529)
Supplement: Supplementary file 1 [file cancers-18-02529-s001.zip › cancers-4461028-supplementary.pdf]

Supplementary Material

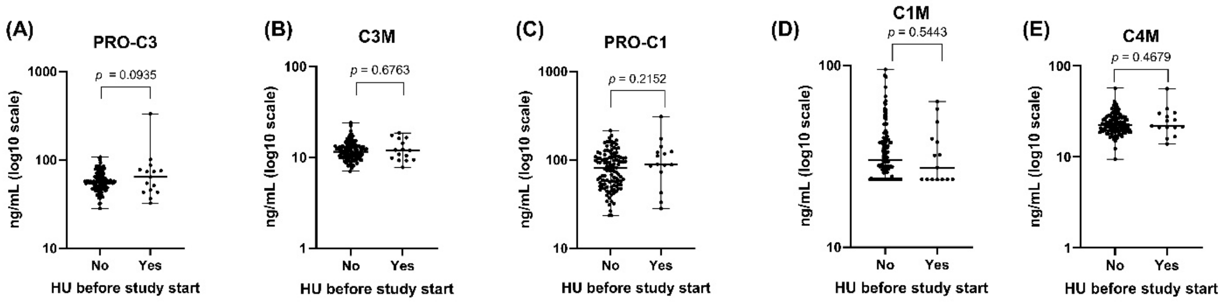

Figure S1. Serum PRO-C3 (**A**), C3M (**B**), PRO-C1 (**C**), C1M (**D**), and C4M (**E**) levels in patients with MPNs ($n = 130$) divided into treatment-naïve patients ($n = 115$) and patients that received HU before study start ($n = 15$). Data points are plotted with geometric mean. Statistical differences were analyzed using an unpaired t-test.

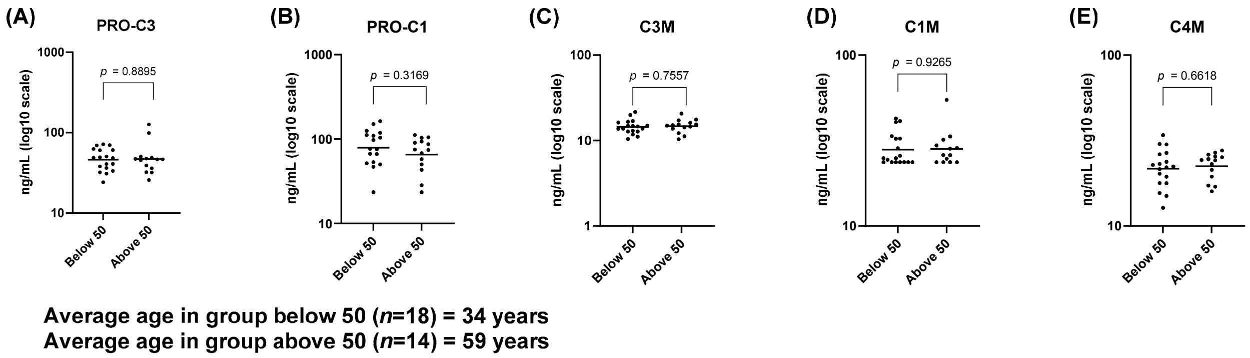

Figure S2. Serum PRO-C3 (**A**), PRO-C1 (**B**), C3M (**C**), C1M (**D**), and C4M (**E**) levels in healthy individuals ($n = 32$) divided into two age groups - one group consisting of individuals with an age below 50 years ($n = 18$, average age = 34 years) and one group with an age of more than 50 years ($n = 14$, average age = 59 years). Data points are plotted with geometric mean. Statistical differences were analyzed using an unpaired t-test.
